# Hormonal predictors of the lean phenotype in humans

**DOI:** 10.17305/bb.2025.12209

**Published:** 2025-04-02

**Authors:** Mohamed Badie Ahmed, Abdella M Habib, Saif Badran, Abeer Alsherawi, Fatima Saoud Al-Mohannadi, Sherouk Essam Elnefaily, Atalla Hammouda, Graeme E Glass, Ibrahem Abdalhakam, Abdul-Badi Abou-Samra, Suhail A Doi

**Affiliations:** 1 Department of Population Medicine, College of Medicine, QU Health, Qatar University, Doha, Qatar; 2 Plastic Surgery Department, Hamad General Hospital, Hamad Medical Corporation, Doha, Qatar; 3 Department of Basic Medical Sciences, College of Medicine, QU Health, Qatar University, Doha, Qatar; 4 Division of Plastic and Reconstructive Surgery, Washington University School of Medicine, Saint Louis, MO, USA; 5 College of Medicine, QU Health, Qatar University, Doha, Qatar; 6 Faculty of Pharmacy, Cyprus International University, Nicosia, North Cyprus; 7 Division of Craniofacial Surgery, Department of Plastic Surgery, Great Ormond Street Hospital for Children NHS Foundation Trust, London, UK; 8 Qatar Metabolic Institute, Academic Health System, Hamad Medical Corporation, Doha, Qatar

**Keywords:** Clinical obesity, adipokines, gut hormones, pancreatic hormones, body contouring surgery, fat mass regulation, amylin, gastric inhibitory polypeptide, GIP, leptin, spexin

## Abstract

Clinical obesity is characterized by excessive fat accumulation and an increased risk of numerous associated comorbidities. Adipose tissue secretes leptin and other adipokines, which play key roles in regulating energy balance, glucose homeostasis, and body fat mass. Recently, incretin and pancreatic hormones have also been shown to influence these processes. However, the regulatory mechanisms and interactions among these hormones are not yet fully understood. This study investigates hormonal predictors of the lean phenotype (in terms of total body fat) in patients undergoing body contouring surgery, with or without prior bariatric surgery. This prospective quasi-experimental study included patients who underwent body contouring procedures at Hamad General Hospital between January 2021 and December 2023. Patients were assessed at three time points: before surgery, 2–3 weeks post-surgery, and 6–10 weeks post-surgery. Body composition and hormone levels were measured, and statistical analyses—including descriptive statistics and logistic regression models—were used to examine trends and predict the lean phenotype. Among the hormones analyzed, amylin showed a significant association with the lean phenotype while increasing leptin, GIP and spexin levels negatively modulated the amylin effect. History of bariatric surgery weakly predicted the lean phenotype after adjusting for leptin and gut hormone levels. A margins plot demonstrated the interactions between amylin, spexin, GIP, and leptin levels that collectively predicted the probability of exhibiting the lean phenotype. These findings highlight amylin, GIP, leptin, and spexin as key hormonal predictors of fat mass, underscoring the critical role of gut hormones and adipokines in determining body fat distribution and the lean phenotype in humans.

## Introduction

Clinical obesity is a chronic disease characterized by the buildup of excessive fat deposits, posing serious effects on health and increasing the risk of developing multiple comorbidities [1]. Adipose tissue, in addition to being an efficient energy resource, is considered an endocrine organ that secretes various hormones known as adipokines, which play a major role in maintaining energy balance and glucose homeostasis. Excess adipose tissue has been associated with insulin resistance (IR), defined as a decreased response of insulin receptors to normal insulin levels, leading to insulin hypersecretion, which has been linked to several comorbidities, including metabolic syndrome, predisposing patients to cardiovascular and metabolic dysfunction–associated steatotic liver disease [2]. Despite the known impact of adipokines on metabolism and glucose homeostasis, the regulation and interactions of these hormones with other signaling molecules, such as incretin and pancreatic hormones, are complex and not fully understood [3]. Recently, researchers have gained interest in incretin and pancreatic hormones due to their impact on glucose homeostasis and weight regulation, complementing the established roles of pancreatic hormones. Understanding the interactions among these adipokines and pancreatic and incretin hormones remains of high importance and warrants further investigation [3].

Adipokines represent a group of hormones secreted by adipocytes that play a major role in metabolism. Of particular importance is leptin, a critical adipokine that plays a key role in fat mass regulation [4–6]. It is mainly secreted from adipocytes, and its plasma concentration increases in proportion to body fat mass. Circulating leptin penetrates the blood–brain barrier to exert its functions in the central nervous system, specifically in the mediobasal part of the hypothalamus [7].

In addition to adipokines, gut-derived hormones such as incretins are key regulators of metabolism and energy balance. The primary incretin hormones, glucagon-like peptide-1 (GLP-1) and glucose-dependent insulinotropic polypeptide (GIP), are secreted from intestinal enteroendocrine cells and enhance glucose-dependent insulin secretion from pancreatic β-cells [8]. Beyond their well-established role in glycemic regulation, GLP-1 receptor agonists (GLP-1 RAs) exert direct effects on adipose tissue, contributing to reductions in fat mass through multiple mechanisms. GLP-1 signaling influences adipose metabolism by modulating lipolysis, adipogenesis, and thermogenesis. Furthermore, GLP-1 signaling in the hypothalamus suppresses orexigenic pathways while activating anorexigenic circuits, leading to reduced caloric intake, which indirectly facilitates fat loss. Tirzepatide, a dual GLP-1/GIP receptor agonist, has demonstrated superior efficacy in promoting weight loss and improving glycemic control in patients with type 2 diabetes and obesity, potentially due to additive or synergistic effects on lipid metabolism and adipose tissue remodeling [9, 10].

Given that incretins exert their effects primarily on pancreatic β-cells, it is important to consider other hormones secreted by these cells that contribute to metabolic regulation. Insulin, the key hormone for glucose homeostasis, is co-secreted with amylin [11]. Amylin plays a major role in inhibiting glucagon secretion from α-cells, slowing gastric emptying, and promoting satiety. The interactions and mechanisms linking these hormones remain of great interest and are not fully understood [12].

Patients undergoing body contouring surgery, with or without a history of bariatric surgery, present a unique model to explore the interplay between body fat mass and hormone expression. Examining hormone levels in the same patients before and after the removal of large volumes of fat enables us to investigate how changes in fat mass influence the regulation of adipokines and gut hormones. These findings may offer valuable insight into how these hormones predict and maintain a lean phenotype in humans. Thus, we aim to assess the interplay between these hormone levels and total body fat percentage in patients pre- and post-body contouring surgery who may or may not have had prior bariatric surgery.

## Materials and methods

### Study population

We investigated patients who underwent body contouring surgeries at the Department of Plastic Surgery at Hamad General Hospital during the period from January 2021 to December 2023. In this prospective quasi-experimental study design, subjects were prospectively followed up at three time points: within one week before surgery, within 2–3 weeks after surgery, and within 6–10 weeks after surgery, to assess variables of interest. These time points were selected to capture immediate as well as delayed changes in hormonal profile after surgery. The inclusion criteria included patients undergoing surgical subcutaneous fat removal (SSFR), also known as body contouring surgery, such as abdominoplasty, lower body lift, or thigh lift (thighplasty), with a body mass index (BMI) ≥18 and age ≥18 years. The exclusion criteria included patients who underwent bariatric surgery less than 18 months prior to body contouring surgery, patients with co-morbidities (except diabetes not on pharmacotherapy) or diabetic nephropathy, patients undergoing body contouring surgeries in areas other than the abdomen or thigh, patients older than 65 years, or patients with a BMI over 35. Informed consent was obtained from all study participants prior to inclusion in the study protocol.

### Assessment of body composition

Body composition was assessed at the defined time points (before and after the surgery) using a Tanita body composition analyzer (DC-360 P), which employs bioelectrical impedance analysis (BIA) technology to provide a detailed full-body composition analysis [13]. The measured variables were weight, body fat percentage, body fat mass, BMI, fat-free mass, estimated muscle mass, total body water, visceral fat rating (VFR), and basal metabolic rate (BMR). Tanita works by sending low and safe electrical signals from four metal electrodes; the signal passes faster through water (hydrated muscle) and meets resistance when passing through fat tissue. The signal outcomes are calculated using scientifically validated Tanita equations to create the final report [14].

### Lean phenotype

In this study, body fat percentages measured using Tanita were divided into tertiles. Subjects in the lowest tertile were defined as the lean phenotype group, highlighting the distinction in body fat mass.

### Oral glucose tolerance test (OGTT)

Fasting glucose levels were measured in the morning after at least 8 h of fasting; then, a 75-g glucose test was performed with plasma glucose measurements taken at 15, 30, 45, 60, and 120 min using a fast multi-assay analyser (Analox-GL5).

### Glycemic indices

Homeostasis model assessment was performed for each subject at the defined two time points (before and after the surgery) using the University of Oxford HOMA2 calculator, which estimates steady-state beta cell function (%B) and HOMA insulin sensitivity (%S) as percentages of a normal reference population [15].

### Body contouring surgeries

Subjects who met the inclusion criteria underwent abdominoplasty, lower body lift, and/or thigh lift surgeries performed according to standard procedures. All surgeries were carried out by expert surgeons in the department.

### Hormonal measurements

Plasma samples were collected at the recruitment center by the recruitment staff, aliquoted, and stored at −70 ^∘^C until further analysis. At the time of analysis, samples were transferred to an adjacent laboratory within the center for hormonal assay. The multiplex panel contained nine hormones (gastric inhibitory polypeptide [GIP], glucagon-like peptide-1 [GLP-1], pancreatic peptide [PP], PYY, amylin, leptin, insulin, C-peptide, and secretin), and only those selected for analysis in this study are reported. These hormones were assessed using EMD Millipore’s MILLIPLEX^®^ Human Metabolic Hormone Panel V3. Using Luminex xMAP technology, this kit enables the simultaneous analysis of the aforementioned analytes in human serum, plasma, tissue/lysate, and culture supernatant samples. In addition, spexin and liver-expressed antimicrobial Peptide 2 (LEAP2) were assessed using ELISA kits manufactured by Abbexa Ltd. Samples were assayed in duplicates in a single assay to exclude inter-assay variations. Intra-assay variations were less than 10%. The MILLIPLEX^®^ Human Metabolic Hormone Panel V3 assay exhibits no or negligible cross-reactivity between the antibodies for each analyte and other analytes in the panel, ensuring high specificity for the target hormones. The spexin ELISA kit is optimized for detecting native spexin, offering a sensitivity of 46.9 pg/mL and a detection range of 78.13–5000 pg/mL. Similarly, the LEAP2 ELISA kit is designed for detecting native LEAP2, with a sensitivity of <0.07 ng/mL and a detection range of 0.156–10 ng/mL.

### Sample size

Sample size calculations were not performed because they require knowledge of the true effect in the study, which is always unknown—not only before but also after the study is conducted—and which, if known, would make conducting the study unnecessary [16]. Furthermore, post hoc assessments of power were not performed because they are deeply problematic (e.g., they are irrelevant, typically biased, and have large sampling variation) and thus were not calculated [17–20]. Instead, in this paper, we included all participants who were available within the time frame of the study.

### Ethical statement

The study received institutional review board (IRB) approval from Medical Research Center at Hamad Medical Corporation under reference MRC-01-20-466.

### Statistical analysis

Descriptive statistics regarding patient demographics and variables of interest were reported by time points (preoperative and postoperative). Differences between the time points were calculated for each variable. Trends in hormonal parameters of interest were analyzed in regression models using visits (1–3), bariatric status (RYGB/SG/None), and demographic characteristics as covariates. Logistic regression models were used to predict the lower tertile of body fat mass %, which will henceforth be labeled the lean phenotype. The correlational structure of repeated measurements in the same patient over time was addressed using cluster-robust standard errors. To better understand the relationships, a margins plot was created to depict the results indicated by logistic regression. To determine if the study data were consistent with a population model that assumes no effect (tested hypothesis), a *P* value was computed [21]. The exact *P* value is reported and indicates the degree of significance (divergence) of the estimated effect from the tested hypothesis, had it been the source of the study data. Results in the interval *P* < 0.05 were labeled ‘statistically significant (divergent)’ [21]. To assess clinical benefit, the point estimate and its 95% uncertainty interval (95% UI, formerly known as the 95% confidence interval) for potentially data-generating test hypotheses were reported, along with an assessment of the practical importance of the study result. All analyses were conducted using Stata Version 17 (StataCorp, College Station, TX, USA).

## Results

### Participants’ characteristics

Subjects who attended visits 1, 2, and 3 were 34, 22, and 27, respectively. BMI, fat % (Tanita), history of bariatric surgery, and hormone levels (leptin, spexin, GLP-1, GIP, PP, amylin, LEAP2) were compared across the three visits (Table 1). The gender distribution remained consistent across visits, with females comprising the majority at all time points. BMI values showed slight fluctuations across visits, with no significant differences observed. Fat percentage demonstrated a reduction at visits two and three compared to the baseline visit; however, no statistical significance was detected. Additionally, the patients’ history of bariatric surgery did not show statistically significant differences across the three visits. Median levels of leptin and spexin exhibited fluctuations across visits. Spexin values demonstrated a consistent increasing trend, while leptin levels showed a slight reduction at visit two followed by an increase at visit three. However, these changes were not statistically significant. In contrast, GLP-1, GIP, PP, LEAP2, and amylin levels demonstrated statistically significant variations across visits, with an initial increase at visit two followed by a reduction at visit three, indicating noteworthy changes over time.

**Table 1:** Baseline characteristics of participants at each visit

**Factor**	**Baseline at visit 1 [median (IQR)]**	**Visit 2 [median (IQR)]**	**Visit 3 [median (IQR)]**	***P* value**
N	34	22	27	
*Gender*				
F	27 (79.4%)	16 (72.7%)	22 (81.5%)	0.75
M	7 (20.6%)	6 (27.3%)	5 (18.5%)	
Age	41.5 (37, 47)	35.5 (34, 37)	43 (37, 49)	0.44
BMI	31.6 (26, 33.2)	30.2 (28.4, 33.3)	30.8 (27.2, 33)	0.85
Fat %	37.1 (32.2, 41.9)	34.1 (32, 36)	34.9 (30.9, 39.3)	0.14
HOMA-S%	73.4 (61.2, 82)	67.3 (53.9, 85.3)	66.0 (57.7, 84.9)	0.89
*History of bariatric surgery*				
None	16 (47.1%)	11 (50.0%)	17 (63.0%)	0.38
Gastric bypass	6 (17.6%)	1 (4.5%)	2 (7.4%)	
Sleeve gastrectomy	12 (35.3%)	10 (45.5%)	8 (29.6%)	
Leptin (µg/L)	9.4 (6.2, 18.6)	7.7 (5, 14.9)	11.2 (8.1, 18.1)	0.32
Spexin (pg/mL)	244.3 (144.6, 457.8)	266.1 (134.3, 469.1)	280.9 (141.8, 374.3)	0.95
GLP-1 (pg/mL)	169.8 (99.2, 329.4)	**298.7 (179.2, 539.9)**	123.3 (103, 180.5)	**<0.001**
GIP (pg/mL)	89 (32.7, 186.5)	**194.8 (136.7, 229.1)**	37.2 (27.1, 56.1)	**<0.001**
PP (pg/mL)	88 (50, 144.3)	**140.3 (56.7, 253.5)**	62 (36.6, 99.7)	**0.02**
LEAP2 (ng/mL)	4.4 (0.9, 11.7)	**11.6 (10.4, 14.2)**	0.8 (0.7, 1.2)	**<0.001**
Amylin (pg/mL)	10.1 (0.0, 22.4)	**17.8 (9.2, 36)**	10.1 (6.6, 13.1)	**0.03**

N: Sample size; F: Female; M: Male; IQR: Inter quartile range; GIP: Gastric inhibitory polypeptide; GLP-1: Glucagon-like peptide-1; PP: Pancreatic peptide; LEAP2: Liver expressed antimicrobial peptide 2.

**Table 2:** Predictors of the lean phenotype using logistic regression

**Lower tertile fat%**	**OR**	***P* > |z|**	**95% uncertainty interval**
Visit			
1	1 (base)		
2	1.377	0.733	0.218, 8.681
3	1.540	0.522	0.411, 5.775
History of bariatric surgery			
No	1 (base)		
BP	3.660	0.308	0.302, 44.361
SL	1.250	0.754	0.311, 5.025
Amylin (pg/mL)	1.098	**0.031**	1.009, 1.196
GIP (pg/mL)	0.989	0.052	0.978, 1.000
Leptin (ug/L)	0.894	**0.026**	0.811, 0.987
Spexin (pg/mL)	0.997	0.105	0.994, 1.000
Constant (baseline odds)	1.110	0.914	0.167, 7.384

*Goodness of fit AUC ═ 0.856; McFaddens R^2^ ═ 0.320: Goodness of link ascertained via linktest in Stata. Base refers to the reference category in the analyses. GIP: Gastric inhibitory polypeptide.

### Predictors of lean phenotype

**Figure 1.**
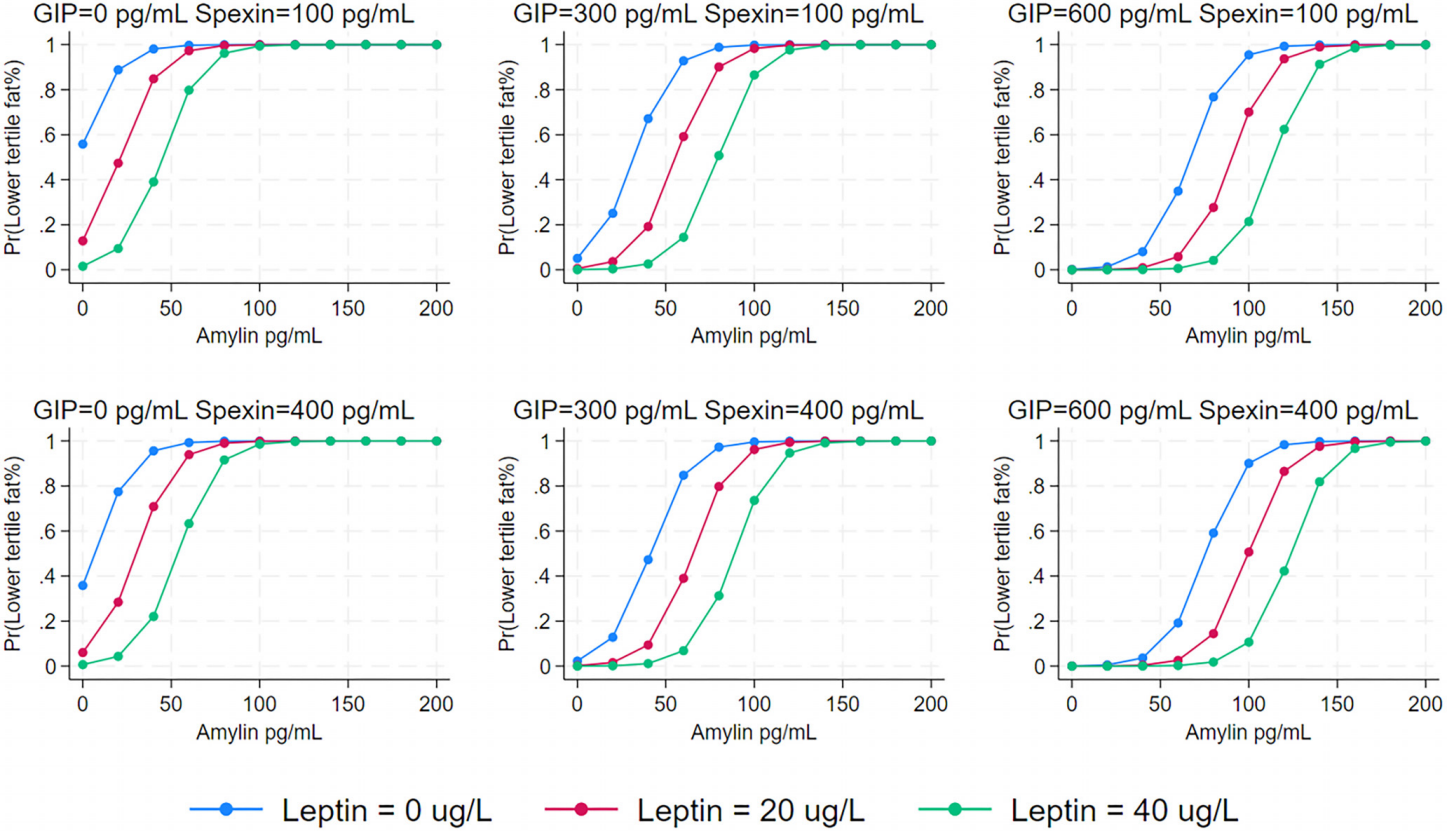
Margins plot with the probability of having low fat% (lower tertile) as a function of plasma amylin (pg/mL) level across two spexin (pg/mL) levels (100 and 400), three GIP (pg/mL) levels (0, 300, 600) and three leptin (ug/L) levels (0, 20, 40).

**Figure 2.**
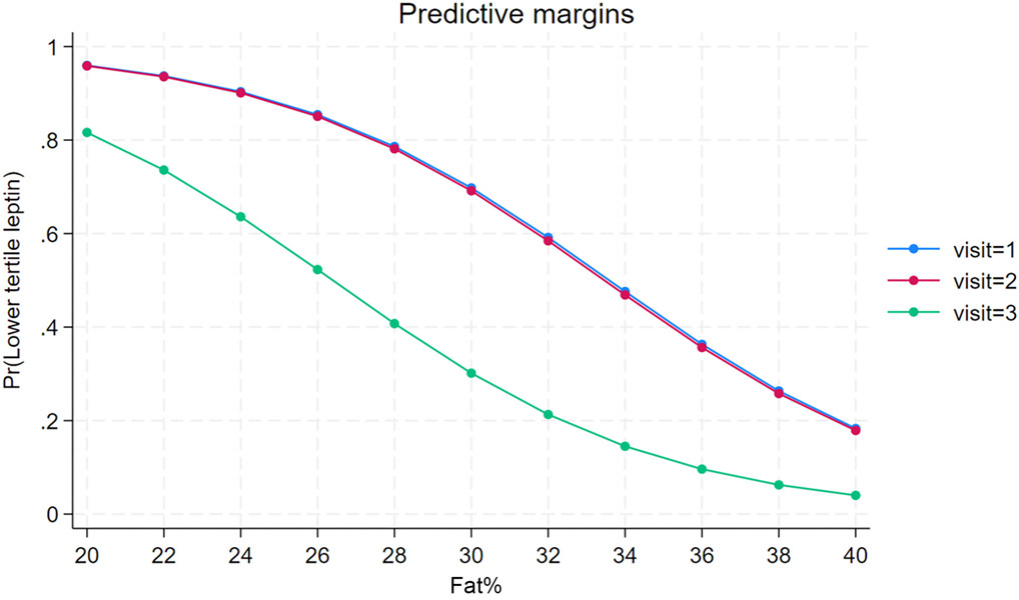
Margins plot with the probability of having low Leptin (ug/L) levels (lower tertile) as a function of fat % across the three visits.

When predictors of lean phenotype were analyzed using logistic regression (Table 2), there was a clinically significant negative association of lean phenotype with plasma leptin (*P* ═ 0.026), GIP (*P* ═ 0.052), and spexin (*P* ═ 0.105) levels. Although some of the *P* values do not reach the threshold for statistical significance, they are low enough to imply a statistical trend, which remains clinically relevant. For each unit increase in leptin, the odds of the lean phenotype decreased by approximately 10%. On the other hand, there was an increase in the likelihood of the lean phenotype with higher plasma amylin levels and a history of bariatric surgery, after accounting for the impact of leptin and gut hormones (GIP and amylin). A margins plot demonstrated that the probability of lean phenotype was mainly predicted by amylin, and the other hormones served to increase the threshold for the amylin effect on fat mass percentage (Figure 1). The only predictor of leptin sensitivity (a proxy for lower tertile leptin levels) was fat mass percentage, with greater fat mass being associated with an increase in plasma leptin—interpreted as a decrease in leptin sensitivity [22]. In addition, immediately after SSFR, there was no change in leptin sensitivity with the sudden loss of fat mass, but at visit three, there was a decline in leptin sensitivity (Figure 2).

## Discussion

This study examined the influence of the levels of gut hormones (amylin and GIP) and adipokines (spexin and leptin) on the prediction of the lean phenotype in a diverse cohort of patients undergoing body contouring surgery. While individual hormones, such as leptin, amylin, GIP, and GLP-1 are known to regulate metabolism, the combined influence of these hormones in predicting body fat mass, particularly a lean phenotype, remains poorly understood. Most previous studies have primarily focused on individual hormones in isolation, leaving the combined influence of these hormones unexplored. This study addresses this gap by investigating how total body fat mass influences these hormones and their potential role in predicting a lean phenotype. Body contouring surgery provides a unique model for studying this relationship through pre- and post-surgical comparisons. By examining hormone levels before and after the surgical excision of subcutaneous fat, we aimed to identify dynamic patterns of hormone levels associated with body fat mass reduction.

The results of this study highlight, for the first time, that amylin, GIP, leptin, and spexin robustly predict the lean phenotype in these participants. Rising levels of amylin and declining levels of all other hormones predicted the lean phenotype. These results suggest that a combination of gut hormones (amylin and GIP) and adipokines (spexin and leptin) are associated with the lean phenotype in humans. While we demonstrate associations, we cannot be certain that the prediction of body fat through hormonal levels in plasma is indicative of cause and effect. However, a look at the literature provides compelling evidence that these hormones play a critical role in body fat regulation and phenotype.

First, it has been reported that amylin is associated with lipolysis in humans. Amylin, a hormone produced in pancreatic β-cells, is well known for its role in regulating food intake and body weight [23–25]. In animal studies, administration of amylin has shown a significant reduction in food intake in a dose-dependent manner, with reductions of over 30% lasting up to 24 h without compensatory hyperphagia [26–28]. This was also demonstrated when an amylin receptor antagonist was injected into rats, resulting in an increase in food intake [29, 30]. Furthermore, amylin administration has been shown to reduce body weight and decrease fat deposition [31–33]. Conversely, opposite findings were observed when an amylin receptor antagonist was injected into rats [34].

Second, several clinical studies have reported the effects of the amylin analog pramlintide on weight control. In diabetic patients (both type 1 and type 2) undergoing insulin therapy, pramlintide treatment resulted in a significant reduction in body weight [35–39]. Similar results were seen in a randomized controlled trial conducted on obese subjects not on insulin therapy, where pramlintide treatment for 16 weeks without concomitant lifestyle intervention led to a significant weight reduction (3.7 ± 0.5%, *P* < 0.001; 3.6 ± 0.6 kg, *P* < 0.001) and a decrease in waist circumference [40]. A long-acting amylin analog (AM833) has shown promise for obesity treatment, with a dose-dependent progressive decrease in body weight of 6%–10.8% over 26 weeks [41]. Given the strong evidence that amylin plays a critical role in body weight regulation, it is plausible that the increased levels of amylin observed in the lean phenotype group contribute to the observed phenotype through mechanisms, such as reduced food intake, delayed gastric emptying, and enhanced satiety [28]. However, sustained long-term fat reduction with amylin is less compared to other therapies such as GLP-1 receptor agonists. Our results suggest that amylin’s effect may depend on favorable levels of GIP, spexin, and leptin, indicating that the interaction among these hormones could be critical for achieving optimal body fat reduction.

Third, it has been reported that GIP had a strong negative relationship with the threshold at which the amylin effect was seen. This aligns with data suggesting that GIP antagonism induces weight loss. Preclinical studies have shown that inhibiting GIP receptors improves insulin sensitivity and reduces obesity [42, 43]. Wild-type mice fed a high-fat diet developed GIP hypersecretion, extreme fat deposition, and IR, while mice lacking GIP receptors were protected from these effects [42]. Another study investigated the effect of chemical ablation of GIP receptors on aspects of obesity-related diabetes [43]. Receptor ablation led to a significant reduction in glucose and insulin levels in response to feeding and improved insulin sensitivity. In addition, it helped correct obesity-related islet hypertrophy and β-cell hyperplasia. Interestingly, chronic administration of a GIP receptor agonist has shown similar effects to a GIP receptor antagonist due to receptor desensitization on adipocytes [44, 45]. This desensitization likely occurs through receptor internalization and degradation pathways, reducing GIP signaling over time. Recent studies indicate that human GIP receptor desensitization involves internalization and downregulation, whereas rodent models may follow alternative pathways, including second messenger-dependent kinases [46]. Current clinical data support this hypothesis, as Tirzepatide, a dual GLP-1 and GIP receptor agonist, has shown superior efficacy in both glycemic control and weight loss compared to other approved medications [9, 47]. This effect is thought to result from the synergistic actions of GLP-1 and GIP, with chronic GIP agonist exposure promoting receptor downregulation, mimicking aspects of GIP antagonism while enhancing insulin sensitivity and fat metabolism [9, 46].

Fourth, leptin also had a similar effect to GIP in terms of increasing the threshold at which amylin began to predict the lean phenotype. Leptin, a hormone secreted by adipose tissue, plays a vital role in maintaining energy homeostasis. Higher leptin levels are associated with greater leptin resistance, a marker of the obese state [48, 49]. Preclinical and clinical studies have shown that leptin and amylin exert synergistic effects on weight loss. In rats, the concurrent administration of leptin and amylin had an additive effect on food intake suppression and weight loss [50, 51]. In human studies, co-administration of recombinant human leptin and the amylin analog pramlintide resulted in a mean weight loss of 12.7 ± 0.9% (11.5 ± 0.9 kg), significantly higher than with either treatment alone [50]. This suggests that restoring leptin sensitivity enhances the fat-reducing effect of amylin. Notably, studies show that amylin improves leptin signaling in the hypothalamus, particularly within the ventromedial hypothalamus, reducing hypothalamic inflammation and promoting sustained weight loss [52].

Our findings, therefore, suggest that the fat-reducing effect of amylin is negatively modulated by increases in leptin, GIP, and spexin, thus impairing the effect of amylin. In a state of extreme obesity, leptin resistance—characterized by impaired hypothalamic signaling, neuroinflammation, and receptor desensitization—may reduce the ability of exogenous leptin to enhance fat metabolism [53]. This impaired signaling may explain why there could be diminished synergy with amylin in obesity [54, 55]. In severely obese rats, amylin effectively reduced body weight and fat mass, but the addition of leptin provided no additional benefit, suggesting that leptin resistance cannot be overcome by exogenous leptin. Notably, caloric restriction-induced weight loss did not restore the leptin effect and may only serve to enhance the amylin effect [55], with the diminished response perhaps reflecting chronic leptin receptor desensitization or altered hypothalamic signaling pathways after a state of extreme obesity. However, contradictory results have been reported, suggesting that the interaction between these hormones is influenced by factors, such as body weight status, leptin sensitivity, and specific dose regimens [54]. These findings highlight the complexity of the amylin–leptin relationship and underscore the need for further investigation to clarify the underlying mechanisms [50].

**Figure 3.**
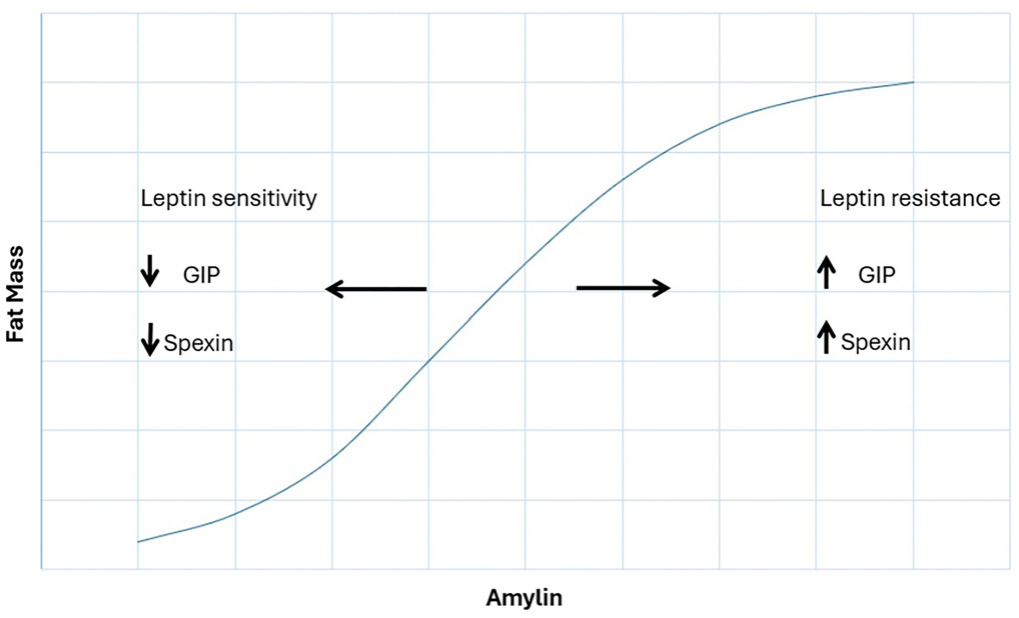
This diagram illustrates the relationship between amylin levels and fat mass, highlighting key factors that modulate this relationship based on our findings.

This study provides a novel exploration of the interplay among gut and pancreatic hormones, adipokines, and their prediction of the lean phenotype in the context of body contouring surgery-induced changes in fat mass. The study’s strength lies in its diverse cohort with varying body fat mass, which enhances the generalizability of the findings. The use of robust statistical models, including logistic regression and cluster robust standard errors, further strengthens the validity of the results by accounting for repeated measures and potential covariates. Cluster robust standard errors help minimize bias related to correlated data within the same patient across multiple time points, ensuring more reliable variance estimates. Despite these strengths, the observational nature of the study limits its ability to establish causality. This raises the possibility of reverse causation or residual confounding. Furthermore, the relatively small sample size, particularly in the post-surgery group, may reduce the statistical power to detect subtle differences, though the associations found were strong despite the sample size. The variability in hormonal levels and the potential confounding factors associated with surgical procedures—such as surgical stress, postoperative recovery, and medication use—add additional complexity to the interpretation of results. Future studies with larger sample sizes and longitudinal designs are necessary to validate these findings and clarify the directionality of the observed associations. Incorporating additional time points and capturing long-term post-surgical hormonal dynamics may provide more comprehensive insights into the complex relationship between these hormones and body fat regulation.

## Conclusion

This study demonstrates a strong association between plasma amylin and the lean phenotype. The modulation of amylin’s fat-regulatory effects may be negatively influenced by increases in plasma levels of leptin, GIP, and spexin; together, these interactions strongly predict the lean phenotype (Figure 3). The lack of predictive value for GLP-1 in this model suggests that GLP-1-related weight loss may involve indirect mechanisms, possibly through the modulation of hormones such as amylin or leptin. These findings underscore the importance of hormonal interactions in understanding and managing obesity and related metabolic conditions. Future research should focus on confirming these associations in larger longitudinal cohorts and elucidating the underlying mechanisms to better establish causality, which can then inform more targeted therapeutic interventions.
